# Looking for Ties with Secret Agendas During the Pandemic: Conspiracy Mentality is Associated with Reduced Trust in Political, Medical, and Scientific Institutions – but Not in Medical Personnel

**DOI:** 10.5334/pb.1086

**Published:** 2022-05-17

**Authors:** Kenzo Nera, Youri L. Mora, Pit Klein, Antoine Roblain, Pascaline Van Oost, Julie Terache, Olivier Klein

**Affiliations:** 1 Center for Social and Cultural Psychology, Université Libre de Bruxelles, BE; 2 Fonds de la Recherche Scientifique, BE; 3 Psychological Sciences Research Institute, Université Catholique de Louvain, BE

**Keywords:** COVID-19, trust, conspiracy mentality, conspiracy theories, populism, power, public health measures

## Abstract

In a preregistered research, we examined the relationships between conspiracy mentality (i.e., the individual susceptibility to endorse conspiracy theories, Bruder et al., 2013) and trust in three actors of the COVID-19 crisis: 1) Political institutions, 2) scientific and medical institutions, and 3) the medical personnel. While the two former groups have played a direct or indirect role in decisions related to public health measures, the latter has not. We expected all these relationships to be negative and mediated by the belief that the pandemic is instrumentalized by authorities to pursue secret agendas. In a study conducted with Belgian (*N* = 1136) and French (*N* = 374) convenience samples, conspiracy mentality negatively predicted trust in political institutions, and trust in scientific and medical institutions. These relations were partly mediated by belief that the pandemic is instrumentalized by authorities. In addition, distrust in political, medical and scientific institutions were highly and positively correlated, suggesting that these groups may be viewed as part of a same supra-ordinate category – the “Elites”. By contrast, we found a small negative relationship between conspiracy mentality and trust in the medical personnel in the Belgian sample, but not in the French sample. Trust in the medical personnel was unrelated to the belief that the pandemic is instrumentalized, and only weakly related to distrust in political institutions. This suggests that individuals with a susceptibility to believe in conspiracy theories may not have a propensity to distrust all actors involved in the management of the pandemic, but only those directly or indirectly tied to decisions pertaining to public health measures.

The management of the COVID-19 pandemic is arguably one of the greatest public health challenges the world has faced over the last century. In most countries, governments took exceptionally stringent measures to contain the spread of the virus (e.g., lockdown, mandatory face masks, social distancing measures, …). These decisions were partly taken based on discussions with members of medical and scientific institutions who assessed the efficacy, costs and benefits of these measures. Finally, a third category of actors, the medical personnel (i.e., doctors and nurses), took care of COVID-19 patients. They had to work under extreme pressure, as many hospitals were overwhelmed by COVID-19 admissions. In later stages of the pandemic, the medical personnel were also in charge of administering vaccine shots to the population.

Trust may be defined as “a psychological state comprising the intention to accept vulnerability based upon the positive expectations of the intentions or behavior of another” (Rousseau et al., 1998, p. 395). Accepting to put oneself in such a state of vulnerability carries the risk of being deceived by those who are endowed with one’s trust (Evans & Krueger, 2009; Warren, 2018). Nevertheless, trust in institutions is crucial to the functioning and development of democratic societies (Mari et al., 2021; Warren, 2018). It may be especially true in a crisis situation such as the COVID-19 pandemic, where the population must cooperate to achieve collective goals (e.g., reduce physical contacts, achieve a high vaccination coverage). In such a context, it is vital to understand the mechanisms susceptible to undermine trust in the actors involved in the management of the crisis.

Belief in conspiracy theories (CT) – explanations of events relying on the concerted action of groups acting in secrecy (Keeley, 1999) – might play a significant role in the population’s distrust of governments, scientific and medical institutions, and medical personnel. While past contributions have examined the relationships between CT beliefs and trust in institutions in the context of COVID-19 (Banai et al., 2021; Bruder & Kunert, 2021; De Coninck et al., 2021; Pummerer et al., 2022), no research has examined if this relationship might differ for the medical personnel – who did not take part in decisions pertaining to the management of the pandemic. Thus, to refine the understanding of the relationship between CT beliefs and trust in times of COVID-19, we propose to distinguish between political institutions, medical and scientific institutions, and the medical personnel in their relationship with individuals’ generic susceptibility to believe in CTs – namely, conspiracy mentality (Bruder et al., 2013; Imhoff & Bruder, 2014; Moscovici, 1987).

While political authorities decided the public health measures, medical and scientific institutions also played a role in designing the measures, as they provided an external expertise to inform political decisions. In France and Belgium, groups of scientists were constituted for this purpose (the GEMS – formerly GEES – in Belgium, and the *Conseil Scientifique Covid-19* in France). These groups, therefore, could be viewed as having exerted much political power during the pandemic. By contrast, most of the medical personnel did not directly take part in political decisions pertaining to the management of the pandemic. Since conspiracy mentality is associated with negative attitudes and distrust towards powerful groups (Imhoff & Bruder, 2014; Imhoff & Lamberty, 2018; 2020a), distinguishing between different actors of the COVID-19 pandemic based on their respective political power appears relevant. However, both conspiracy mentality and specific CT beliefs are associated with paranoid tendencies towards outgroups (e.g., through the path of collective narcissism, Bertin et al., 2021; Cichocka, 2016; Cichocka et al., 2016), paranoia (Imhoff & Lamberty, 2018), and interpersonal distrust (De Coninck et al., 2021; Jovančević & Milićević, 2020). Thus, even though these relationships might differ in strength, we expected conspiracy mentality to negatively predict trust in these three groups of actors.

In addition, we propose that the negative relationships between conspiracy mentality and trust in these actors may be mediated by the belief that the pandemic is instrumentalized by authorities to pursue an unlawful, secret agenda. Indeed, since conspiracy mentality is associated with a propensity to suspect hidden realities and secret agendas (e.g., Moscovici, 1987), we expected conspiracy mentality to elicit such suspicions in the context of the COVID-19 pandemic.

## Conspiracy Beliefs and Trust in Actors Managing the COVID-19 Crisis

CT beliefs^1^ have a deleterious impact on public health. For instance, CT beliefs reduce vaccination intentions (e.g., Allington et al., 2021; Bertin et al., 2020; Jolley & Douglas, 2014) and are associated with risky sexual behaviors (Bogart & Thorburn, 2005; Brooks et al., 2018; Jolley & Jaspal, 2020). In the context of the COVID-19 pandemic, CT beliefs are associated with reduced compliance with social distancing measures (Biddlestone et al., 2020; Bierwiaczonek et al., 2020; Bruder & Kunert, 2021; Imhoff & Lamberty, 2020b; Marinthe et al., 2020, Study 2; Pummerer et al., 2022). Interestingly, Marinthe et al. (2020) showed that CT beliefs were associated with increased endorsement of non-normative social distancing behaviors (e.g., stop going out to public places), which at the time of the study were more extreme than the measures advised by authorities (e.g., stop shaking hands or kissing). Bruder and Kunert (2021) also reported nuanced findings showing that CT beliefs were related to reduced compliance to social distancing measures, but not reduced compliance to hygiene related recommendations.

### Conspiracy Beliefs and Trust in Political Authorities

The fact that conspiracy mentality might reduce trust in actors managing the COVID-19 pandemic is predictable based on past literature. Indeed, CT beliefs are known to be associated with reduced trust in political institutions (e.g., Einstein & Glick, 2015; Mari et al., 2021; Nera et al., 2021). Similarly, CT beliefs are associated with negative attitudes towards various powerful groups (Imhoff & Bruder, 2014) and cultural minorities (e.g., Bilewicz et al., 2013; Uenal et al., 2021), which can be viewed as indicators of distrust towards these groups. In the context of the COVID-19 pandemic, CT beliefs are associated with reduced trust in political authorities (Banai et al., 2021; Bruder & Kunert, 2021; Pummerer et al., 2022). Given the tight relationship between conspiracy mentality and specific CT beliefs, we hypothesize that this finding will be replicated in the context of our studies, and that conspiracy mentality will predict distrust in political authorities managing the COVID-19 crisis.

### Conspiracy Beliefs and Trust in Scientific and Medical Institutions

There is large body of evidence showing negative relationships between CT beliefs and trust in science. Indeed, CT beliefs are typically associated with anti-science attitudes (Goertzel, 2010; Lamberty & Imhoff, 2018; Lewandowsky et al., 2013). For example, CT beliefs play a prominent role in the rejection of climate science (Bertin et al., 2021; Goertzel, 2010; Uscinski et al., 2017). Even when CT beliefs are unrelated to vaccination, they are associated with a rejection of vaccine science (Bertin et al., 2020; Lewandowsky et al., 2013). In the context of the COVID-19 pandemic, scholars have argued that the relationship between CT beliefs and reduced compliance with public health measures may be to a large extent mediated by a reduced trust in science (Plohl & Musil, 2021). In a similar vein, Uscinski et al. (2020) have shown that a key correlate of COVID-19 CT beliefs was the rejection of information coming from scientific experts.

As for medical institutions, CT beliefs are also associated with lesser endorsement of conventional (i.e., evidence-based) medicine, and increased support for non-conventional therapeutic approaches that are perceived as less powerful (e.g., acupuncture or holistic medicine, Lamberty & Imhoff, 2018, see also Oliver & Wood, 2014). Moreover, CT beliefs have been shown to be associated with reduced trust in the healthcare system – even though the relationship was weaker than for trust in authorities and trust in science (Bruder & Kunert, 2021). Hence, we expect that conspiracy mentality will predict reduced trust in medical and scientific institutions – as they provide science-based justification for the public health measures and advise political authorities.

### Conspiracy Beliefs and Trust in the Medical Personnel

The potential negative association between CT beliefs and trust in the medical personnel is less straightforward. Indeed, contrary to political and scientific institutions, the medical personnel did not take part in the development and application of public health measures. Thus, this group may not be construed as holding as much power as political, medical, and scientific institutions. They are therefore less likely to elicit negative attitudes (Imhoff & Bruder, 2014; Imhoff & Lamberty, 2020a) and distrust (Imhoff et al., 2018; Lamberty & Imhoff, 2018) among CT believers. This relative powerlessness, in combination with the fact that the expression “medical personnel” emphasizes that these people are those who directly take care of patients, may among CT believers strengthen the subjective distinction between these actors and those who, even though some of them might be doctors, may not *really* care about the wellbeing of the population (Vignaud & Salvadori, 2019).

However, research suggests that both conspiracy mentality and specific CT beliefs are associated with reduced interpersonal trust (De Coninck et al., 2021) and paranoia (for a meta-analysis, see Imhoff & Lamberty, 2018). In the context of COVID-19, Jovančević and Milićević (2020, Serbian sample) also reported data showing that CT beliefs about COVID-19 having been fabricated in a lab are associated with reduced interpersonal trust. This suggests that conspiracy mentality may be associated with decreased trust towards just any group – including the medical personnel taking care of COVID-19 patients. We therefore expect the relationship between conspiracy mentality and trust in the medical personnel to be negative.

## Belief that the Pandemic is Instrumentalized as a Mediator

One of the defining features of CTs is that they are based on the rejection of official narratives (i.e., an “official version” of events broadcasted by authorities or a scientific consensus, e.g., Guillon, 2018; Keeley, 1999; Lantian et al., 2016). Indeed, CTs typically assert that the actions of powerful groups (such as the government, politicians, or intelligence agencies) are ultimately determined by malevolent secret agendas (Bruder et al., 2013; Imhoff & Bruder, 2014). In line with this view, Moscovici (1987, 2020) has proposed that conspiracy mentality is structured by representations of concealed realities and forbidden knowledge – as conspirators actively seek to maintain the population in ignorance. Despite the conceptual limitations of such a dispositional approach to belief in CTs (Nera et al., 2021; Sutton & Douglas, 2020), it is well established that individuals believing in some CTs tend to endorse others, even when they are unrelated (Goertzel, 1994; Sutton & Douglas, 2020; Wood et al., 2012). Since measures of conspiracy mentality adequately capture individuals’ endorsement of a broad range of CT beliefs (Frenken & Imhoff, 2021), conspiracy mentality can still be viewed as a measurement of individuals’ general susceptibility to endorse CTs.

Hence, in the specific context of the pandemic, individuals with a general tendency to believe in CTs may have the sense that the management of the pandemic (e.g., public health measures, vaccination campaigns) covers up a secret and unlawful agenda. Indeed, in the course of the pandemic, many CTs asserted that public health measures aiming at mitigating the COVID-19 pandemic were an excuse to further control and monitor the population, for instance through the use of vaccines and 5G (e.g., Bruns et al., 2020; Nguyen & Catalan-Matamoros, 2020). In this view, COVID-19 might or might not be a hoax (Imhoff & Lamberty, 2020b), it might be a bioweapon or a natural virus; it may have been created by the Chinese government (e.g., Bertin et al., 2020; Romer & Jamieson, 2020), pharmaceutical companies (e.g., Bertin et al., 2020), or mysterious “dark forces” (Imhoff & Lamberty, 2020b). However, these various CTs share a reliance on the idea that the *pandemic is instrumentalized by authorities to pursue a secret, unlawful agenda*.

In turn, the perception that the COVID-19 pandemic is instrumentalized is likely to reduce trust in the aforementioned actors managing the crisis (i.e., political, scientific and medical institutions, and the medical personnel). Indeed, if the pandemic is suspected to be instrumentalized by authorities to pursue a secret and malevolent agenda, the actors involved in the management of the crisis are likely to be perceived as untrustworthy.

This may be especially plausible for members of political, scientific and medical institutions, as these groups hold positions of power in society and may be conflated in the general category of the “Elites” among CT believers. Indeed, powerful groups tend to be perceived as part of the same category (i.e., the corrupted elites/“Establishment”) in populist ideologies (Mudde & Kaltwasser, 2017), and such ideologies are associated with CT beliefs (Castanho Silva et al., 2017; Eberl et al., 2021; van Prooijen, 2018). However, the belief that the pandemic is instrumentalized may also predict reduced trust in the medical personnel, who, despite their lower status, may be perceived as parties to the conspiracy. Indeed, while research on CTs tend to construe these beliefs as narratives opposing a malevolent outgroup to a victim ingroup (e.g., van Prooijen & van Lange, 2014), a more fine-grained examination of CT believers’ view of society reveals that one can distinguish between accomplices of the conspiracy (i.e., groups enacting the conspiracy, for example by maintaining the population in ignorance) and the conspirators (Franks et al., 2017). Thus, the medical personnel might be perceived as subordinates of the conspirators.

As such, we expect the negative relationship between conspiracy mentality and trust in actors managing the crisis (especially political institutions, as well as scientific and medical institutions) to be mediated by the belief that the pandemic is instrumentalized by authorities to pursue a secret and unlawful agenda.

## Overview of the research

Given the magnitude of the challenge posed by the COVID-19 pandemic and the documented resistance to its management by authorities, it is crucial to investigate the variables that influence trust in the different actors at the forefront of the crisis, and the mechanisms underlying these relations. Our research took place during the first wave of COVID-19 (April 2020). During this period, both Belgium and France were under lockdown. It examines the extent to which conspiracy mentality – which captures a general tendency to believe in CTs (Bruder et al., 2013) – predicts a decreased trust in three key actors of the COVID-19 crisis: political institutions, scientific and medical institutions, and the medical personnel.

**Figure 1:**
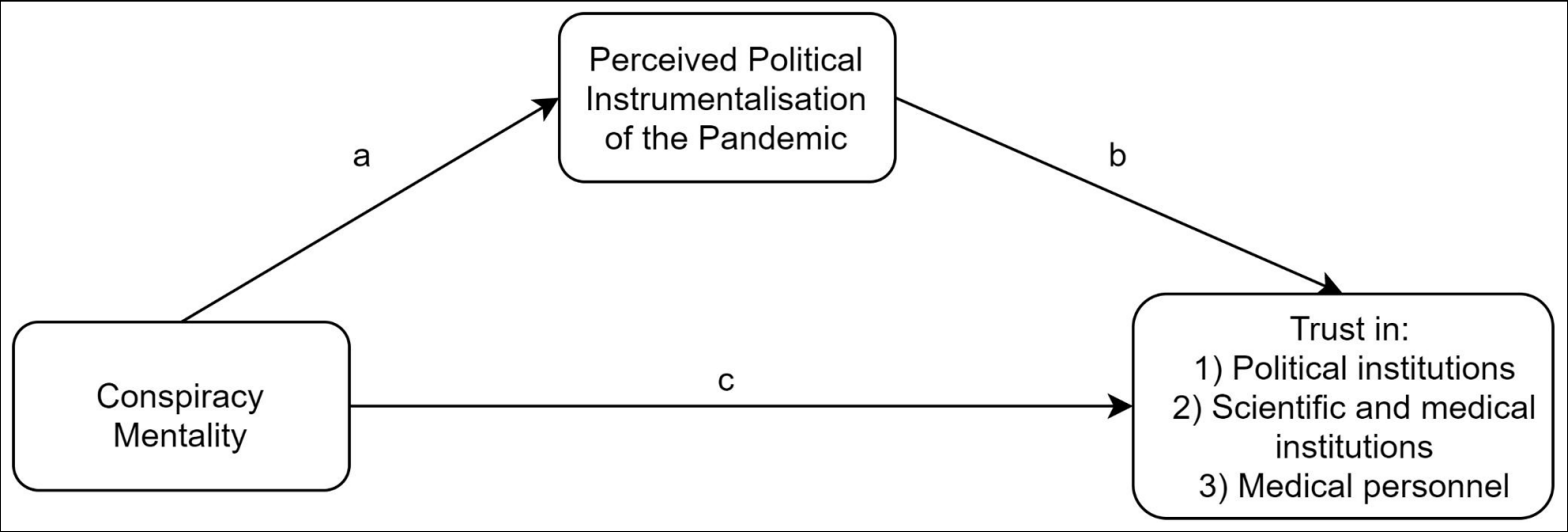
Mediation model for trust in political institutions scientific and medical institutions, and medical personnel.

We predict that conspiracy mentality would be negatively associated with trust in these three actors of the crisis (H_1_, H_2_, H_3_; path *c* on Figure 1). Since conspiracy mentality is associated with the sense that authorities pursue secret and malevolent agendas, we expect that these relations will be mediated by the belief that the COVID-19 pandemic is instrumentalized by power holders (H_1b_, H_2b_, H_3b_; paths *a* and *b* on Figure 1).^2^ The theoretical model is formalized in Figure 1 and was identically applied to the three dependent variables.

To test the robustness of the proposed model, we sought to test it in two distinct (yet relatively close) cultural contexts: Belgium and France. Since the institutions and the actors managing the crisis were different in these two countries, the data were analyzed separately.

This study was pre-registered prior to data analysis. Hypotheses and planned analyses are available on the Open Science Framework: https://osf.io/5v7kh. Datasets^3^ and *R* script are also available on https://osf.io/hy2vn/.

## Method

### Participants and procedure

We conducted two cross-sectional online studies in April 2020, relying on convenience samples. Data collection was conducted in Belgium (Study 1a) and France (Study 1b) using two online surveys (in French) that were disseminated via email, online social networks (snowball sampling among the acquaintances of the authors), and separate sponsored Facebook advertisements. The ad read as follows: “Our study (15 minutes) is about the perception of politics and solidarity movements during the COVID-19 crisis. This questionnaire is entirely anonymous. Thank you in advance for your participation!”. Thus, the questionnaire was not explicitly about the topic of conspiracy theories and trust. The scales used in this article were included in a larger questionnaire examining, among other variables, individuals’ causal attribution regarding the origin of COVID-19, past voting behaviors and future voting intentions, and emotions experienced during the lockdown. As indicated in the ad, the study took approximately 15 minutes to complete. Anyone above 18 years old and residing in Belgium (Study 1a) or France (Study 1b) could participate.

For Study 1a, the sample consisted of 1130 Belgian participants (753 women, 359 men, 4 “a gender not listed above”, 14 did not disclose). For Study 1b, the sample consisted of 368 French participants (264 women, 102 men, 0 “a gender not listed above” and 2 participants did not disclose). Participants were on average 46.26 years old (*SD* = 14.37) in Belgium, and 47.5 years old (*SD* = 14.45) in France. Among Belgian participants, 36.5% had a Master’s degree (36.6% in the French sample) and 77% attended higher education (76% in the French sample). Note that answering the questions was not mandatory. Therefore, analyses are associated with varying degrees of freedom due to missing values.

The convenience sampling was not weighed in order to better represent the population in terms of gender, age or education levels. As a consequence, descriptive estimates should not be taken as unbiased estimators of the population.

### Measures

#### Conspiracy Mentality

Conspiracy mentality was measured using the 5-item Conspiracy Mentality Questionnaire (Bruder et al., 2013)^4^, an 11-point Likert scale ranging from 0% (certainly not true) to 100% (certainly true). Participants were asked to express their agreement with the following statements: “*Many very important things happen in the world, which the public is never informed about*”, “*politicians usually do not tell us the true motives for their decisions*”, “*government agencies closely monitor all citizens*”, “*events which superficially seem to lack a connection are often the result of secret activities*” and “*there are secret organizations that greatly influence political decisions*”. It yielded a Cronbach’s alpha of .88 for the Belgian sample, and .90 for the French sample.

#### Belief in instrumentalization of the pandemic

To measure the mediator, participants were asked to rate their level of agreement with 3 items related with the instrumentalization of the pandemic, on a 5-point Likert scale ranging from “*Totally disagree”* (1) to *“Totally agree”* (5): “*The pandemic is (or will be) instrumentalized by some politicians in order to pass reforms that otherwise would have been unacceptable*”, “*Some leaders want to use the pandemic to pursue political agendas that have nothing to do with the public health situation*” and “*The pandemic might be used to vote restrictions on freedoms that would have been unthinkable otherwise*”. These items were designed for this study. The scale yielded a Cronbach’s alpha of .85 for the Belgian sample, and .96 for the French sample.

Since conspiracy mentality conceptually encompasses a susceptibility to suspect secret agendas, we carried out confirmatory factor analyses to check if conspiracy mentality and belief in the instrumentalization of the pandemic were distinct constructs. We compared the fit of a single factor structure to the fit of a two-factor structure using the ‘lavaan’ *R* package (Rosseel, 2012) and the “anova” function. In the Belgian sample, the fit of the two-factor structure, *χ^2^*(19) = 450, *p* < .001, CFI = 0.91, TLI = 0.86, SRMR = 0.06, RMSEA = 0.14, was superior to the fit of a single factor structure – which was insufficient, *χ^2^*(20) = 1492, *p* < .001, CFI = 0.68, TLI = 0.56, SRMR = 0.12, RMSEA = 0.26. The fit difference was statistically significant, *χ^2^ difference* = 899, *p* < .001. In the French sample, the fit of the two-factor structure, *χ^2^*(19) = 107.05, *p* < .001, CFI = 0.97, TLI = 0.95, SRMR = 0.05, RMSEA = 0.12, was also largely superior to the fit of the single factor structure, *χ^2^*(28) = 2790.84, *p* < .001, CFI = 0.69, TLI = 0.56, SRMR = 0.28, RMSEA = 0.38. This difference was also significant, *χ^2^ difference* = 778.34, *p* < .001. Thus, as expected, belief in the instrumentalization of the pandemic and conspiracy mentality appear to be distinct constructs.

#### Trusts in actors of the COVID-19 crisis

Trust was measured with a scale designed for this study. Participants were asked the following: “Please report your personal trust in the following institutions”. Four items measured trust in political institutions (Politicians”, “the Belgian/French government”, “the Belgian/French parliament”, “political parties”), yielding a Cronbach’s alpha of .91 for the Belgian sample, and .90 for the French sample. Two items measured trust in scientific and medical institutions (“the healthcare system”, “scientific and medical experts”). It yielded a Cronbach’s alpha of .55 for the Belgian sample, and 62 for the French sample.^5^ Finally, trust in the medical personnel was measured with a single item (“the medical personnel”). Participants reported their trust on a 5-point Likert scale anchored from “*No trust at all”* to “*Total trust”*.^6^

We examined if the hypothesized three-factor structure of trust towards actors of the crisis returned a satisfactory fit. The Confirmatory Factor Analysis returned a good fit for the Belgian sample, *χ^2^*(12) = 69.6, *p* < .001, CFI = 0.985, TLI = 0.974, SRMR = 0.016, RMSEA = 0.065, as well as the French sample, *χ^2^*(12) = 68.5, *p* < .001, CFI = 0.957, TLI = 0.924, SRMR = 0.031, RMSEA = 0.112.

#### Sociodemographics variables

Participants reported their age, gender (woman/man/a gender not listed above/prefer not to disclose; in the analyses, gender as a covariate was converted into a binary man/woman variable). Participants also reported their perceived probability of having caught COVID-19, which was measured with one item on a 5-point Likert scale (ranging from “*You are certain you did get the coronavirus”* to “*You are certain you did not get the coronavirus”*): *“Even if you have not been diagnosed, to what extent do you think you have had the virus?”*.

## Results

Descriptive estimates as well as correlations for Study 1a and Study 1b are displayed in Tables 1 and 2, respectively. As pre-registered, for each dependent variable, to control potential confounds, we conducted hierarchical regression models controlling for gender, age, and perceived probability of having caught COVID-19 at Step 1. At Step 2, conspiracy mentality was added as an independent variable. We carried out pathway mediation analyses with the ‘lavaan’ R package (Rosseel, 2012, see online supplements for analyses scripts). In line with the suggestions of Yzerbyt et al. (2018), we do not report single indexes for the significance of indirect effects. Rather, we separately report paths a and b in the mediation model. Both effects being significant means that the indirect effect is also significant.

**Table 1:** Means, standard deviations, and correlations with confidence intervals for the Belgian sample.


VARIABLE	*M*	*SD*	1	2	3	4	5	6

1. Conspiracy mentality	5.58	2.35						

2. Belief in instrumentalization	4.03	0.91	.44** [.39, .48]					

3. Trust in political institutions	2.05	0.84	–57** [–.61,–.53]	–.44** [–.49, –.39]				

4. Trust in medical and scientific institutions	3.63	0.80	–.43** [–.48, –.38]	–.30** [–.35, –.25]	.50** [.45, .54]			

5. Trust in the medical personnel	4.46	0.65	–.08** [–.14, –.02]	–.04 [–.10, .02]	.13** [.07, .19]	.41** [.36, .45]		

6. Age	46.26	14.37	.12** [.07, .18]	–.01 [–.07, .05]	–.16** [–.21, –.10]	–13** [–.19, –.08]	.03 [–.03, .09]	

7. Perceived risk of having caught COVID	2.64	0.91	.03 [–.03, .09]	.05 [–.01, .11]	–.05 [–.11, .01]	–.08** [–.14, –.02]	–.05 [–11, .01]	–.10** [–.16,–.04]


*Notes*: *M* and *SD* are used to represent mean and standard deviation, respectively. Values in square brackets indicate the 95% confidence interval for each correlation. * *p* < .05. ** *p* < .01.

**Table 2:** Means, standard deviations, and correlations with confidence intervals for the French sample (Study 1b).


VARIABLE	*M*	*SD*	1	2	3	4	5	6

1. Conspiracy Mentality	6.27	2.51						

2. Belief in instrumentalization	4.00	1.23	.40** [.31, .48]					

3. Trust in political institutions	2.00	0.89	–.58** [–.64, –.51]	–46** [–.54, –.38]				

4. Trust in medical and scientific institutions	3.28	0.95	–.39** [–.47, –.30]	–.30** [–.39, –.20]	.57** [.50, .63]			

5. Trust in the medical personnel	4.51	0.66	.01 [–.09, .11]	–.01 [–.11, .09]	.13* [.03, .23]	.23** [.13, .33]		

6. Age	47.50	14.45	.10* [.00, .20]	.07 [–.04, .17]	–.08 [–.18, .03]	–.19** [–.29, –.09]	.08 [–.02, .18]	

7. Perceived risk of having caught COVID	2.58	1.04	.04 [–.06, .15]	.02 [–.09, .13]	–.12* [–.22, –.01]	–.08 [–.19, .02]	–.03 [–.13, .08]	–.11* [–.21, –.00]


*Notes*: *M* and *SD* are used to represent mean and standard deviation, respectively. Values in square brackets indicate the 95% confidence interval for each correlation. * *p* < .05. ** *p* < .01.

### Confirmatory analyses

#### Study 1a – Belgian sample

**Figure 2:**
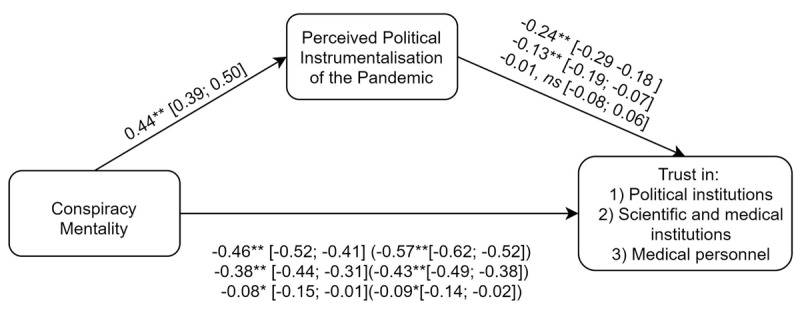
Mediation analyses (Study 1a, Belgian sample). *Note*. Each line corresponds to the *beta* regression coefficients for a different dependent variable (in descending order: political institutions, scientific and medical institutions, and the medical personnel). Between round brackets are total effects. Between square brackets are 95% confidence intervals.

H_1_ predicted a negative linear relationship between conspiracy mentality and trust in political institutions. Congruent with this hypothesis, the total effect of conspiracy mentality on trust in political institutions was significant in the mediation model, *β* = –0.57, 95% CI [–0.62, –0.52], *z* = –21.87, *p* < .001 (Figure 2, c+[a*b], 1^st^ line). Congruent with H_1b_, this relationship was partly mediated by perceived instrumentalization of the pandemic. Indeed, belief in political instrumentalization of the pandemic was significantly predicted by conspiracy mentality, *β* = 0.44, 95% CI [0.39, 0.50], *z* = 15.42, *p* < .001 (Figure 2, path a) and, in turn, belief in political instrumentalization of the pandemic negatively predicted trust in political institutions, *β* = -0.24, 95% CI [–0.29, –0.18], *z* = –8.63, *p* < .001 (Figure 2, path b, 1^st^ line). The direct effect of conspiracy mentality on trust in political institutions remained significant, *β* = –0.46, 95% CI [–0.52, –0.41], *z* = –16.64, *p* < .001 (see Figure 2, path c, 1^st^ line).

Note that among the covariates, only age was a significant predictor for trust in political institutions, *β* = –0.11, 95% CI [–0.16, –0.06], *z* = –4.27, *p* < .001. Perceived probability of having caught COVID-19 was not a significant predictor, *β* = 0.03, 95% CI [–0.02, 0.09], *z* = 1.16, *p* = .247, and neither was gender, *β* = –0.04, 95% CI [–0.14, 0.07], *z* = –0.69, *p =* .492.

H_2_ predicted a negative linear relation between conspiracy mentality and trust in medical and scientific institutions. Congruent with the hypothesis, the total effect of conspiracy mentality on trust in medical and scientific institutions was significant, *β* = –0.43, 95% CI [–0.49, –0.38], *z* = –15.30, *p* < .001 (Figure 2, c+[a*b], 2^nd^ line). In support of H_2b_, we found evidence for a mediation by belief in instrumentalization of the pandemic. Indeed, belief in the instrumentalization of the pandemic significantly predicted trust in medical and scientific institutions, *β* = –0.13, 95% CI [–0.19, –0.07], *z* = –4.33, *p* < .001 (Figure 2, path b, 2^nd^ line).^7^ The direct effect of conspiracy mentality remained significant, *β* = –0.38, 95% CI [–0.44, –0.31], *z* = –12.03, *p* < .001 (Figure 2, path c, 2^nd^ line).

Among the covariates, only age and perception of having caught COVID were significantly associated with trust in medical and scientific institutions, respectively, *β* = –0.10, 95% CI [–0.15, –0.04], *z* = –3.40, *p* = .001, and *β* = –0.07, 95% CI [–0.13, –0.02], *z* = –2.65, *p* = .008.

H_3_ predicted a negative linear relation between conspiracy mentality and trust in the medical personnel. Corroborating H_3_ the total effect of conspiracy mentality on trust in the medical personnel was significant, *β* = –0.09, 95% CI [–0.15, –0.02], *z* = –2.69, *p* = .007 (Figure 2, c+[a*b], 3^rd^ line). H_3b_ was not corroborated, as we found no evidence of a mediation by belief in political instrumentalization of the pandemic. Specifically, belief in political instrumentalization of the pandemic did not significantly predict trust in the medical personnel, *β* = –0.01, 95% CI [–0.08, 0.06], *z* = –0.22, *p* = .827 (Figure 2, path b, 3^rd^ line). Given the absence of mediation, the direct effect of conspiracy mentality on this dependent variable remained significant, *β* = –0.08, 95% CI [–0.15, –01], *z* = –2.33, *p* =.020 (Figure 2, path c, 3^rd^ line), supporting H3. We observed no significant effect of the covariates on trust in the medical personnel.

#### Study 1b – French sample

Just like in Study 1a, we conducted hierarchical regressions (controlling for gender, age, and perceived probability of having caught the COVID-19) for each dependent variable, namely, trust in political institutions, trust in medical and scientific institutions, and trust in the medical personnel. At Step 2, conspiracy mentality was added as an independent variable.

**Figure 3:**
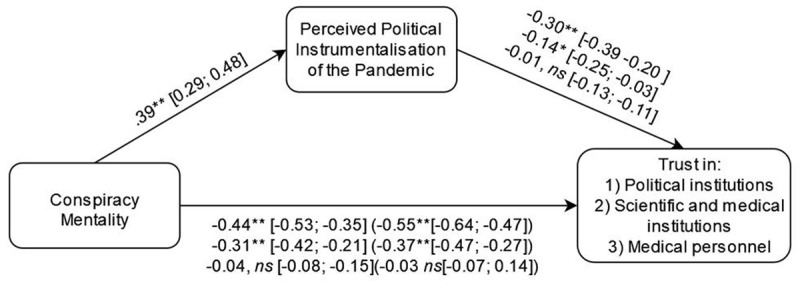
Mediation analyses (Study 1b, French sample). *Note*: Each line corresponds to the *beta* regression coefficients for a different dependent variable (In the following order: political institutions, scientific and medical institutions, and the medical personnel). Between round brackets are total effects. Between squared brackets are 95% confidence intervals.

Congruent with H_1_ and results of Study 1a, the total effect of conspiracy mentality on trust in political institutions was significant, *β* = –0.55, 95% CI [–0.64, –0.47], *z* = –12.21, *p* < .001 (Figure 3, c+[a*b], 1^st^ line). In corroboration of H_1b_, we observed a partial mediation: Conspiracy mentality predicted belief in political instrumentalization of the pandemic, *β* = 0.39, 95% CI [0.29, 0.48], *z* = 7.73, *p* < .001 (Figure 3, path a), and in turn, belief in political instrumentalization negatively predicted trust in political institutions, *β* = –0.30, 95% CI [–0.39, –0.20], *z* = –6.29, *p* < .001 (Figure 3, path b, 1^st^ line). The direct effect of conspiracy mentality on trust in political institutions remained significant, *β* = –0.44, 95% CI [–0.53, –0.35], *z* = –9.44, *p* < .001 (Figure 3, path c, 1^st^ line). We observed no significant effect of the covariates on trust in political institutions.

Congruently with H_2_ and Study 1a, the total effect of this model was significant, *β* = –0.37, 95% CI [–0.47, –0.27], *z* = –7.34, *p* < .001 (Figure 3, c+[a*b], 2^nd^ line). As expected from H_2b_, this relationship was significantly mediated by belief in the instrumentalization of the pandemic. Indeed, belief in the instrumentalization of the pandemic negatively predicted trust in the medical and scientific institutions, *β* = –0.14, 95% CI [–0.25, –0.03], *z* = –2.55, *p* = .011 (Figure 3, path b, 2^nd^ line). The direct effect of conspiracy on trust in medical and scientific institutions remained significant, *β* = –0.31, 95% CI [–0.25, –0.03], *z* = –5.83, *p* < .001 (Figure 3, path c, 2^nd^ line).

Amongst covariates, only age yielded a significant effect on trust in medical and scientific institutions, *β* = –0.14, 95% CI [–0.24, –0.05], *z* = –2.86, *p* = .004.

Against H_3_, the total effect of conspiracy mentality on trust in the medical personnel was not significant, *β* = 0.03, 95% CI [–0.07, 0.14], *z* = 0.61, *p* = .545 (Figure 3, c+[a*b], 3^rd^ line). Indeed, we found no evidence for an effect of belief in instrumentalization of the pandemic on trust in the medical personnel, *β* = –0.01, 95% CI [–0.13, 0.11], *z* = –0.16, *p* = .871 (Figure 3, path b, 3^rd^ line), which is inconsistent with the mediation postulated by H_3b_. The direct effect of conspiracy mentality on trust in the medical personnel remained non-significant, *β* = 0.04, 95% CI [–0.08, 0.15], *z* = 0.62, *p* = .535 (Figure 3, path c). None of the covariates had a significant influence on trust in the medical personnel.

### Exploratory Analyses

We carried out Fisher’s r-to-z tests to compare the strength of the association between conspiracy mentality and trust in the different actors of the pandemic.^8^ In the Belgian sample, conspiracy mentality was negatively correlated with trust in political institutions, *r* = –.57, *p* < .001, and trust in medical and scientific institutions, *r* = –.43, *p* < .001. A Fisher’s r-to-z test revealed that conspiracy mentality was significantly more related to trust in political institutions than to trust in scientific and medical institutions, *z* = –5.70, *p* < .001. Similarly, conspiracy mentality was more related to trust in medical and scientific institutions than to trust in the medical personnel (*r* = –.08, *p* < .05), *z* = –9.49, *p* < .001.

In the French sample, we found similar results. Conspiracy mentality was related to both trust in political institutions, *r* = –.58, *p* < .001, and trust in scientific and medical institutions, *r* = –.39, *p* < .001. Conspiracy mentality was significantly more related to trust in political institutions, *z* = –8.21, *p* < .001. Trust in scientific and medical institutions was more related to conspiracy mentality than trust in the medical personnel (*r* = .01, *ns*), *z* = –11.28, *p* < .001. Finally, a trust in the medical personnel in Belgium and in France did not significantly differ, *z* = –1.50, *p* = .067.

Finally, we tested whether the levels of trust towards political institutions, medical institutions and medical personnel significantly differed. In the Belgian sample, pairwise *t*-tests with Bonferroni corrections for three comparisons revealed statistically significant differences in trust score between political institutions and medical and scientific institutions, *t*(1107) = 64.3, *p* < .001 (*M_Political institution__s_* = 1.93, *SD* = 0.97; *M_Medical and scientific institutions_* = 3.51, *SD* = 1.18); political institutions and medical personnel, *t*(1107) = 81.3, *p* < .001 (*M_medical personnel_* = 4.83, *SD* = 0.61); medical and scientific institutions and medical personnel, *t*(1107) = 35.0, *p* < .001. Hence, the level of trust towards political institutions was lower than the trust towards scientific and medical institutions, which was lower than the trust towards medical personnel.

In the French sample, pairwise *t*-tests with Bonferroni corrections revealed statistically significant differences in trust scores between political institutions and scientific and scientific institutions, *t*(366) = 28.6, *p* < .001 (*M_Political institutions_* = 1.84, *SD* = 0.96; *M_Medical and scientific institutions_* = 3.12, *SD* = 1.20); political institutions and medical personnel, *t*(366) = 46.4, *p* < .001 (*M_medical personnel_* = 4.78, *SD* = 0.66); medical and scientific institutions and medical personnel *t*(366) = 22.9, *p* < .001. Hence, just like in the Belgian sample, the level of trust towards political institutions was lower than the trust towards medical institutions, which was lower than the trust towards medical personnel.

## Discussion

In both samples, conspiracy mentality (i.e., generic propensity to endorse CTs, Bruder et al., 2013) was a robust negative predictor of trust in political institutions. This replicates past research (Banai et al., 2020; Bruder & Kunert, 2021; Einstein & Glick, 2015; Nera et al., 2021; Pummerer et al., 2020; van Prooijen et al., 2021). Also in line with past research (Bruder & Kunert, 2021; Plohl & Musil, 2021; Uscinski et al., 2020), conspiracy mentality was associated with reduced trust in scientific and medical institutions. Moreover, and also congruent with past research (Bruder & Kunert, 2021), conspiracy mentality was more strongly associated with trust in political institutions than with trust in scientific and medical institutions.

The data was compatible with a mediation by the belief that the COVID-19 pandemic is instrumentalized by authorities to pursue an unlawful agenda. Thus, our results corroborate the idea that conspiracy mentality is robustly associated with the belief that authorities pursue secret agendas (Bruder et al., 2013). Such belief is in turn predictive of distrust of political and public health institutions. This suggests that the distrust of public health and scientific institutions can be partly explained by the perception that these institutions are connected to hidden political agendas.

This finding may be interpreted through the lens of research showing robust relationships between – both specific and generic – CT beliefs and populist attitudes (Castanho Silva et al., 2017; Eberl et al., 2021; van Prooijen, 2018). Indeed, populism relies on the opposition between the “pure people” and the elites, which collapses political institutions, scientific experts, and the media (Mudde & Kaltwasser, 2017; van Prooijen, 2018). Hence, CT believers might perceive scientific and medical institutions as being part of (or at least, controlled by) “the elites”. Besides, the strong correlations between trust in political, scientific and medical institutions (*r*s >= .50) suggests that one’s trust in political authorities is a robust indicator of one’s trust in scientific and medical institutions.

By contrast, regarding the relationship between conspiracy mentality and trust in the medical personnel, we found a weak – yet statistically significant – negative relationship in the Belgian sample, but not in the French sample. This might be explained by the fact that overall, the level of trust in the medical personnel was very high, scoring on average at 4.46 (in Belgium) and 4.51 (in France) on a scale ranging from 1 to 5. Moreover, unlike scientific and medical institutions, trust in the medical personnel was only weakly correlated to trust in political institutions. Thus, among the actors managing the crisis examined in this research, the medical personnel seem to be the only group which is *not* less trusted by individuals with a propensity to believe in CTs. Moreover, neither the belief that the pandemic is instrumentalized by authorities, nor trust in political institutions, were substantially associated with distrust in the medical personnel. This might be explained by the fact that the medical personnel were not involved in political decisions related to public health measures. As a result, CT believers, who tend to infer threat from power (Imhoff & Bruder, 2014; Imhoff & Lamberty, 2020a), may not perceive the medical personnel as a particularly powerful – and therefore, threatening – group in the context of the COVID-19 pandemic.

This finding may be put in perspective with Bruder and Kunert (2021), who found a negative relationship between COVID-19 CT beliefs and trust in the German healthcare system. Technically speaking, the medical personnel are also part of the institutional healthcare system. However, while the healthcare system designates an official institution, the medical personnel designate a collection of people, namely, healthcare professionals. Thus, among CT believers, talking about the “healthcare system” may emphasize the institutional ties of healthcare professionals, and therefore strengthen the sense that this group cannot be trusted. As a result, future research may benefit from distinguishing the healthcare system as an institution, from healthcare workers – professionals taking care of the patients.

These findings can also be put in perspective with the fact that in both samples, we observed that participants trusted scientific institutions more than politicians, and the medical personnel more than scientific and medical institutions. The very high levels of trust in the medical personnel – even among CT believers – may be good or bad news for the management of the pandemic. On the bright side, we found that CT believers do not distrust all groups involved in the management of the pandemic. This would have been a grim scenario – which was plausible considering research showing that CT beliefs are associated with generalized distrust of others (De Coninck et al., 2021; Imhoff & Lamberty, 2018, Jovančević & Milićević, 2020, Serbian sample). Our results suggest that communication with the public could benefit from being presented and promoted by healthcare professionals who take care of COVID-19 patients – rather than as scientific experts, or as representatives of scientific or medical institutions.

On the other hand, in the light of these results, the fact that some members of the medical personnel question the relevance of the public health measures, or even refuse to get vaccinated (RTBF, December 6th, 2021), may constitute an important obstacle to the acceptance of public health measures. Indeed, if members of a social group that elicits strong levels of trust in the population spreads misinformation and CTs pertaining to the pandemic, they may have an important impact on the public’s opinion regarding public health measures. Thus, finding ways to enhance the medical personnel’s trust in authorities and scientific institutions – over and above making vaccination mandatory for the medical personnel (BX1, January 4th, 2022) – appears as an important issue to address.

## Limitations and Future Directions

The first limitation is the cross-sectional nature of the data which does not allow conclusions regarding causality. It is possible, for example, that individuals are drawn to develop a conspiracy mentality because they distrust authorities, and not the other way around. Of course, when dealing with such complex social phenomena, the two causal directions are not exclusive and might reinforce each other. Moreover, the absence of experimental manipulation of the mediator does not enable us to provide a robust test of the mediation hypothesis.

Second, the differential levels of trust expressed should be taken with caution, for two reasons. The most obvious reason is that the reported studies relied on convenience sampling, which does not allow to draw conclusions regarding the opinion of the general population. Second, the data was collected at a time when every evening at 8 P.M., the Belgian population applauded the medical personnel who was struggling with the first wave of COVID-19 (RTBF, March 18th, 2020). Two years later, it is possible that the perception of the medical personnel has changed, notably in relation to the vaccination campaign. Indeed, the data was collected before any vaccine was available. It is possible that after the medical personnel started administering the vaccine shots in the population, CT believers may have – at least partly – changed their minds about this group.

Third, we did not have access to participants’ belonging to one of the considered groups (i.e., political, scientific, medical institutions, and the medical personnel). While we assumed that these groups may be considered as outgroups by participants, we should have ideally controlled for individuals’ belonging to these groups in our analyses. Note that the group categorization processes at stake among medical workers and political figures who spread COVID-19 CTs would be interesting to investigate, as these individuals seemingly distrust their own ingroups.

Finally, we measured trust in medical and scientific institutions as a single construct whose 2-item measurement yielded a relatively low reliability. The reason for this choice was that we sought to measure trust in groups who advised governments regarding public health measures, and these groups are tied to scientific institutions, but also to the healthcare system. Even though alternative analyses with separate items returned similar results, a more fine-grained conceptualization may allow to clearly distinguish the groups that provided scientific advice to authorities in the context of the pandemic.

## Conclusion

Despite these limitations, the current article has contributed to the literature in three ways. First, we have shown that while conspiracy mentality predicted decreased trust in political, medical and scientific institutions – which replicates past findings (e.g. Bruder & Kunert, 2021; Einstein & Glick, 2015; Pummerer et al., 2020) – it was not (or barely) the case for trust in the medical personnel. Thus, our research mitigates the hypothesis that CT believers distrust all the actors involved in the management of the pandemic. The fact that the medical personnel did not directly take part in decisions related to the public health measures might explain this different relationship. The relationships between power perceptions and trust, which have been investigated in past research (e.g., Imhoff et al., 2018; Lamberty & Imhoff, 2018), should be further investigated in the context of the COVID-19 pandemic.

Second, we have shown that trust in the different actors in charge of the crisis varied greatly: Participants reported very low levels of trust towards political institutions, moderate levels of trust towards scientific and medical institutions, and very high levels of trust towards the medical personnel. To our knowledge this finding was not reported in previous research.

Last and relatedly, we have shown that these relationships between conspiracy mentality and political, medical and scientific institutions might be partially mediated by the belief that the pandemic is and/or will be instrumentalized by authorities to pursue secret agendas. Hence, the relationship between conspiracy mentality and trust in science, scientists, and medical institutions may be in part due to the perception that these institutions are tightly related to political authorities – and their secret agendas. This echoes Eberl et al. (2021) who have shown that populist attitudes were associated with distrust in both scientific and political elites, and participates in bridging the gap between research on CT beliefs and trust (Banai et al., 2020; Bruder & Kunert, 2021; Einstein & Glick, 2015; Nera et al., 2021; Pummerer et al., 2020; van Prooijen et al., 2021), and research on CT beliefs and populist attitudes (Castanho Silva et al., 2017; van Prooijen, 2018).

For effective public health campaigns (e.g., encouraging people to get vaccinated against COVID-19), it might be important to consider the salient group memberships of the communicators. For instance, placing the medical personnel at the front stage might be a lead to reduce the distrust of the vaccine-reluctant public.

## Footnotes

Note that many of the findings reviewed in the following sections pertain to correlates of specific CT beliefs (e.g., about vaccination or COVID-19). However, since measures of conspiracy mentality (or similar measurements of individuals’ generic propensity to believe in CTs, e.g., Generic Conspiracist Beliefs, Brotherton et al., 2013) are tightly related to belief in specific CTs, it is likely that these results are valid for conspiracy mentality. Indeed, conspiracy mentality may be related to these various outcomes either directly, or through the path of specific CT beliefs.

In the preregistration, we planned on including a second mediator, perceived dangerousness of COVID-19. Due to theoretical and methodological considerations, this mediator was removed from the analysis. The inclusion of the mediator did not change any of the reported results.

Data files for the two samples are named “COVID_BE.sav” and “COVID_FR.xslx”. The R script used for data analysis is named “COVID19_CMQ_Trusts.R”.

The questionnaire was in French. Here we reported the English version of the items. For the validated French version, see the supplementary materials of Lantian et al. (2016).

Given the relatively low reliability of the scales, we also ran the analyses on the items separately for the healthcare system and scientific and medical experts. For both items, we found the expected direct and indirect effects (see online supplements on the OSF).

The trust towards the National Security Council was pre-registered as a third item for this variable. However, we later disagreed on the nature of this institution, stressing its political role even if its mission is essentially health related. Using an exploratory analysis (Oblimin rotation with minimum residuals extraction), it was loading very similarly on the two dimensions (.50 on the Political factor and .41 on the Health factor). Finally, as this institution does not exist in France, we deviated from pre-registration and decided to remove this item from analysis. This did not change the fit of any factor, nor any of the results.

Path a (prediction of perceived instrumentalization by conspiracy mentality) is identical for all 3 dependent variables. In order to succinctly describe the mediation, it is only reported on its first occurrence (see reported results for H1b).

These tests were carried out on the following website: https://www.psychometrica.de/correlation.html.
